# Editorial: Pathophysiological Mechanisms of Sarcopenia in Aging and in Muscular Dystrophy: A Translational Approach

**DOI:** 10.3389/fnagi.2015.00153

**Published:** 2015-08-13

**Authors:** Luciano Merlini, Paolo Bonaldo, Emanuele Marzetti

**Affiliations:** ^1^Laboratory of Musculoskeletal Cell Biology, Istituto Ortopedico Rizzoli, Bologna, Italy; ^2^Department of Molecular Medicine, University of Padova, Padova, Italy; ^3^Department of Geriatrics, Neurosciences and Orthopedics, Teaching Hospital “Agostino Gemelli”, Catholic University of the Sacred Heart School of Medicine, Rome, Italy

**Keywords:** skeletal muscle, frailty, autophagy, apoptosis, mitochondrial dysfunction, signaling pathways, satellite cells, treatment

Sarcopenia and muscular dystrophy are both characterized by the loss of muscle mass and increased intramuscular fibrosis. The two conditions also share several pathophysiological mechanisms, including mitochondrial dysfunction, increased apoptosis, abnormal regulation of autophagy, decline in satellite cell function, increased generation of reactive oxygen species, and alterations of signaling and stress response pathways. Basic science researchers and clinicians working in the areas of sarcopenia and muscular dystrophy in human and animal models contributed to this research topic (Alway et al., 2014; Calvani et al., 2014; Cesari et al., 2014; De Palma et al., 2014; Gattazzo et al., 2014; Harris-Love et al., 2014; Hepple, 2014; Holland et al., 2014; Holmberg et al., 2014; Jungbluth and Gautel, 2014; Kern et al., 2014; La Rovere et al., 2014; Lim et al., 2014; Malatesta et al., 2014; Marzetti et al., 2014; Merlini et al., 2014; Palmio and Udd, 2014; Pozzobon et al., 2014; Raz and Raz, 2014; Rudolf et al., 2014; Sabatelli et al., 2014; Sakuma et al., 2014; Sanchis-Gomar et al., 2014; Tamaki et al., 2014; Toni et al., 2014; Zulian et al., 2014; Krause, 2015). The aim of this cross-fertilization was to accelerate our understanding of the mechanisms involved in aging and dystrophic sarcopenia and to explore the therapeutic potential of various intervention modalities.

Since its first “official” appreciation back in 1989 (Rosenberg, 1989), sarcopenia has attracted great research interest. The intimate relationship between muscle loss and advancing age, evident across multiple species, has led researchers to consider sarcopenia as a paradigm for exploring the aging process as a whole. Sarcopenia is indeed envisioned as a biomarker of aging, able to distinguish, at the clinical level, biological from chronological age (Fisher, 2004). This view is supported by the association reported between sarcopenia and the length of telomeres in peripheral blood mononuclear cells (PBMCs), a popular biological marker of senescence (Marzetti et al., 2014). This relationship seemed to be driven mainly by the muscle mass, which suggests the existence of a common pathogenic basis for muscle atrophy and telomere attrition. No significant association was found between PBMC telomere length and muscle strength or function parameters, pointing at distinct pathogenic mechanisms underlying the quantitative and qualitative dimensions of sarcopenia.

Decreased number and impaired function of satellite cells, the major stem cell population responsible for skeletal muscle regeneration during adulthood, are considered to be major the mechanisms contributing to sarcopenia (Chakkalakal and Brack, 2012). Along with changes in systemic factors and the environmental cues provided by satellite cells, oxidative stress that characterizes the aged muscle contributes to impairing satellite cells responses to exercise, disuse, and rehabilitation. Some nutritional intervention may improve satellite cell function in aged muscles; however, the effect of nutraceuticals on satellite cell function in the context of sarcopenia is not yet well established. Alway et al. (2014) provided an overview on the contribution of satellite cell dysfunction to sarcopenia. The authors also reviewed data from preclinical studies showing that the administration of specific nutraceuticals (resveratrol, green tea catechins, and β-hydroxy-β-methylbutyrate) may improve satellite cell function, thereby mitigating sarcopenia and promoting muscle recovery after disuse.

Most studies aimed at testing potential therapeutic approaches targeting satellite cells in muscle diseases have been conducted in small mammalian models. Though, our knowledge of muscle regeneration in large animal models, such as dogs, remains relatively limited. La Rovere et al. (2014) investigated the myogenic potential of satellite cells isolated from somite- and presomite-derived muscles of young and aged dogs, as well as from Golden Retrievers affected by muscular dystrophy. Satellite cells obtained from the two canine sources showed different proliferation and differentiation rates. Indeed, those isolated from presomitic-derived muscle showed higher telomerase activity and stronger stem cell potential, whereas satellite cells obtained from somitic-derived muscle expressed early and late myogenic markers, resulting in more efficient cell differentiation. Interestingly, expression profiling of muscle-specific miRNAs revealed a unique epigenetic signature in Golden Retriever muscular dystrophy, suggesting that miR-206 might represent a potential target for therapy.

Satellite cell dysfunction is involved in collagen VI-related myopathies. Collagen VI is an extracellular matrix protein, essential for muscle homeostasis (Bonaldo et al., 1998). Mutations in the gene encoding for collagen VI cause different forms of inherited myopathies and congenital muscular dystrophies. Studies carried out during the last decade in collagen VI null mice provided new insights into the pathomolecular mechanisms of collagen VI-related myopathies, leading to pilot clinical trials in human subjects. In addition to mitochondrial dysfunction and defective autophagy, lack of collagen VI affects muscle regeneration and self-renewal of satellite cells. Gattazzo et al. (2014) investigated the effects of cyclosporin A on muscle regeneration in collagen VI null mice under basal conditions and after cardiotoxin-induced muscle injury. The authors provided evidence that cyclosporin A influenced satellite cell activity and triggered the formation of regenerating fibers. Data obtained on collagen VI null mice showed that, under appropriate administration regimens, cyclosporin A was capable of stimulating myogenesis and aiding in regeneration of injured muscles.

Muscular dystrophies perturb regenerative processes, which causes the premature exhaustion of the satellite cell reservoir due to continuous cycles of degeneration/regeneration (Decary et al., 2000). Pozzobon et al. (2014) provided an overview on the use of fetal-derived stem cells as a new therapeutic approach in muscle diseases. The authors highlight that cells of fetal origin (e.g., cord blood, placenta, amniotic fluid) can be easily obtained without ethical concern and expanded and differentiated in culture; such cells also possess immune-modulatory properties. They also highlight that different types of fetal stem cells of human and mouse origin display myogenic differentiation capabilities.

In recent years, mitochondrial dysfunction has received increasing attention as a possible target for interventions against sarcopenia (Marzetti et al., 2013). Mitochondria are indeed located at the cross-roads of several vital pathways, including energy production, redox homeostasis, cellular quality control, and cell death/survival signaling. As pointed out by Hepple (2014), however, the degree by which advancing age impacts muscular mitochondrial function is probably milder than previously thought. In fact, the procedures for mechanical isolation of mitochondria can themselves induce alterations in the intrinsic function of the organelle. In contrast, assays performed using permeabilized muscle fibers may provide more reliable results because mitochondria are not subjected to artificial mechanical damage and are analyzed within their normal architectural organization in the myofiber. Based on the most recent evidence, Hepple (2014) proposed that dysfunctional mitochondrial autophagy and an increased propensity toward permeability transition may represent relevant therapeutic targets for anti-sarcopenic interventions.

Mitochondrial dysfunction plays a major role in the pathogenesis of collagen VI-related myopathies through a short circuit caused by inappropriate opening of the permeability transition pore. The latter is a high-conductance channel that causes a shortage of ATP production (Bernardi and Bonaldo, 2013). Melanocytes obtained from skin biopsies do not produce collagen VI, yet they bind collagen VI at the cell surface, suggesting that this protein plays a trophic role in healthy muscle (Zulian et al., 2014). Mitochondria of melanocytes of patients with collagen VI-related myopathies display increased size, reduced matrix density, and disrupted cristae, indicative of mitochondrial functional impairment. After inhibition of the F_1_F_0_-ATP synthase with oligomycin, mitochondria underwent anomalous depolarization and showed decreased respiratory reserve capacity. The non-immunosuppressive cyclophilin inhibitor NIM811 prevented mitochondrial depolarization in response to oligomycin in melanocytes from myopathic patients.

Well-functioning autophagy is fundamental for organismal health and longevity (Bonaldo and Sandri, 2013). Excessive activation of autophagy-dependent degradation contributes to muscle atrophy and cachexia, whereas its suppression results in accumulation of protein aggregates and abnormal organelles, leading to myofiber degeneration. Lim et al. (2014) provide a detailed overview of the current knowledge of Pompe disease with a discussion on recent insights into the molecular mechanisms and novel perspectives for therapy. Pompe disease is a lysosomal storage disorder causing progressive accumulation of glycogen-filled lysosomes in several tissues, with cardiac and skeletal muscles being the most severely affected. Lysosomal enlargement was long considered to be the major mechanism causing muscle damage in Pompe disease. However, accumulating evidence indicates that dysfunctional autophagy and accelerated production of lipofuscin inclusions are deeply involved in the onset of muscle damage and interfere with acid alpha-glucosidase delivery during therapy. Several novel therapeutic approaches have recently been tested in mouse models of Pompe disease, including lysosomal exocytosis and manipulation of autophagy.

Along similar lines, De Palma et al. (2014) review recent findings showing that dysfunctional autophagy plays a pathogenic role in muscular dystrophies. The authors highlight the clinical relevance of the recent findings on the role of autophagy and its regulatory signaling pathways in Duchenne muscular dystrophy. They also discuss how modulating autophagy may represent a promising strategy for therapeutic interventions aimed at counteracting muscle wasting in muscular dystrophies.

Jungbluth and Gautel (2014) provided an overview of the clinical, histopathological, and genetic aspects of centronuclear myopathies in the context of key pathogenic mechanisms. Models to study and rescue the affected cellular pathways are now available in yeast, *Caenorhabditis elegans*, Drosophila, zebrafish, mouse, and dog. Defects in membrane trafficking have emerged as a key pathogenic mechanism, in particular aberrant T-tubule formation, abnormalities of triadic assembly, and disturbance of the excitation–contraction machinery. Abnormal autophagy has recently been indicated as another important pathogenic mechanism in different genetic forms of centronuclear myopathies that may be potentially amenable to therapeutic interventions.

The complexity of the cellular mechanisms underlying the pathophysiology of sarcopenia may be deciphered through the use of well-characterized muscle disorders that recapitulate, at least in part, the phenotype of pathological muscle aging. Late-onset autosomal dominant oculopharyngeal muscular dystrophy (OPMD) may well serve that purpose (Raz and Raz, 2014). OPMD is a rare monogenic disorder caused by an expansion mutation in the gene encoding poly-adenylate RNA binding protein1 (PABPN1). Symptoms begin in midlife and progress with advancing age. Ocular and pharyngeal muscles are those most commonly affected at the disease onset. As the disorder progresses, other muscles, especially of the lower limbs, become impaired. The molecular signatures of OPMD muscles show remarkable overlap with sarcopenia. For instance, the accumulation of insoluble protein aggregates, a hallmark of OPMD, has also been observed in aged muscles and attributed to a dysfunctional ubiquitin-proteasome system and defective autophagy. It is worth noting that mitochondrial dysfunction contributes to muscle degeneration in both OPMD and sarcopenia, further supporting the proposition that OPMD may represent a model for accelerated muscle aging.

Sabatelli et al. (2014) observed aggresome-autophagy involvement in a sarcopenic patient with rigid spine syndrome and a p.C150R mutation in the four-and-half LIM domain protein 1 gene (FHL1). The 34-year-old wheelchair-bound woman had an early and progressive rigidity of the cervical spine, marked diffuse muscle wasting, weakness, prominent contractures, and severe respiratory insufficiency. According to a BMI of 17.1, she was underweight; however, her body composition, estimated by DXA, revealed that she was sarcopenic/obese with a marked reduction of lean body mass and a total body fat of 44%. Muscle biopsy showed multiprotein aggregates throughout the cytoplasm and around myonuclei with aggresome/autophagy features and nuclear degradation.

Other pathophysiologic mechanisms of aging and dystrophic sarcopenia were explored by the seven following contributions.

The possibility of considering muscular dystrophy a model of accelerated sarcopenia is supported by the observation that the two conditions exhibit marked defects of the autophagy-lysosome system, likely as a consequence of dysfunctional Akt/mTOR/p70S6K pathway and disruption of serum response factor (SRF)-dependent signaling (Sakuma et al., 2014). Upregulation of myostatin signaling is also commonly observed in both muscular dystrophy and sarcopenia. Remarkably, preclinical studies have shown that pharmacological downregulation of myostatin signaling attenuates the pathological phenotype in sarcopenic rodents and several mouse models of muscular dystrophy.

A large number of studies in *mdx* mice, the rodent model of Duchenne muscular dystrophy, led to the concept that aging exacerbates the dystrophic phenotype of dystrophin-deficient mice. Holland et al. (2014) outline recent data on the age-dependent changes of the senescent *mdx* muscle proteome and discuss these findings in comparison with the proteomic profile of sarcopenic muscle. These comparative muscle proteomics not only confirmed similar perturbations in a number of biochemical processes, but also revealed striking similarities in cellular stress responses between the two conditions.

Muscle denervation is a hallmark of sarcopenia and muscular dystrophy (Deschenes, 2011). During the development of sarcopenia and muscular dystrophy, neuromuscular junctions (NMJs) deteriorate and display altered molecular features. Rudolf et al. (2014) provided an overview of NMJ alterations observed in sarcopenic and dystrophic muscles. The authors also reviewed the current knowledge on the molecular mechanisms underlying NMJ degeneration during aging and in the context of muscular dystrophies. As pointed out by Rudolf et al. (2014), the observation that physical exercise can reverse NMJ abnormalities in the aged muscle opens new venues for the design of treatments targeting the NMJ to rescue muscle mass in sarcopenia and muscular dystrophies.

It is a common notion that the loss of muscle mass and strength follows different trajectories over time, with steeper declines in strength relative to mass (Goodpaster et al., 2006). Findings from Tamaki et al. (2014) provide a convincing explanation for such a phenomenon. The authors elegantly show that qualitative alterations of the peripheral motor system (i.e., reduction of shortening and relaxing velocity of twitch, impaired motor unit recruitment at high stimulation frequencies, and early NMJ fatigability) occur well before the appearance of the sarcopenic phenotype in middle-aged rats. Interestingly, significant decreases in muscle shortening and relaxing velocity during serial twitch contractions, accompanied by type-IIb to type-I fiber shift, were detected in middle-aged rats in the absence of muscle atrophy. These changes may be prodromic to the well-known preferential loss of fast-twitch fibers that characterize the sarcopenic muscle.

Malatesta et al. (2014) provide convincing arguments in favor of a partly common physiopathologic substratum for myotonic dystrophy (DM) and sarcopenia. DM is an autosomal dominant disorder that originates from nucleotide expansions. At the histological level, DM and sarcopenia are characterized by myofiber atrophy, fiber size variability, and centrally located nuclei. The two conditions also share a number of ultrastructural and functional features, including nuclear rearrangement of the RNP-containing domains, likely due to defects in the machinery responsible for pre-mRNA transcription and maturation, and satellite cell dysfunction. Interestingly, both in DM and sarcopenia, muscleblind-like 1 (MBNL1), an alternative-splicing factor, undergoes intranuclear relocation and accumulation, which contributes to hampering the functionality of the whole splicing machinery. This, in turn, reduces nuclear metabolic activity, therefore impairing protein synthesis. Defects in pre-mRNA post-transcriptional pathways are believed to account for the aging-reminiscent muscle phenotype of DM patients and suggest that muscle wasting in DM and sarcopenia may originate from similar mechanisms.

Within the intricate network of mechanisms underlying the pathophysiology of muscular dystrophies, miRNAs certainly play a role (Eisenberg et al., 2009). miRNAs are widespread regulators of gene expression, but little is known about their potential roles in congenital muscular dystrophies. MDC1A is a severe form of congenital muscular dystrophy caused by mutations of the gene encoding laminin α2, a key component of the basal lamina in muscle endomysium. To gain insight into the pathophysiological roles of miRNAs associated with MDC1A Holmberg et al. (2014) analyzed a number of miRNAs in the skeletal muscles of laminin α2 chain-deficient mice and found that expression of muscle-specific miR-1, miR-133a, and miR-206 is deregulated in these muscles. They also demonstrate that plasma levels of muscle-specific miRNAs are elevated in laminin α2 chain-deficient mice and are partially normalized in response to proteasome inhibition. These findings indicate that muscle-specific miRNAs are deregulated in MDC1A and suggest that their plasma levels may represent promising biomarkers for evaluating disease progression.

Several different and partly overlapping muscle degeneration pathways are involved in another group of myopathies characterized by the presence of inclusions, such as hereditary inclusion body myopathy (IBM), IBM with Paget’s disease of bone and frontotemporal dementia (IBMPFD), and *GNE* myopathy (Krause, 2015). Mutations in VCP/p97 responsible for IBMPFD are associated with defective myosin assembly, deregulation of major protein degradation pathways, reduced regeneration capability, and impaired mitochondrial quality control. However, a plethora of mechanisms underlying disease onset in hereditary IBMs remains to be elucidated at the molecular and pathophysiological level.

The five following contributions explored clinical aspects and evaluation methods. Harris-Love et al. (2014) provide preliminary data about the use of real-time augmented feedback for quantitative ultrasound imaging. The authors suggest that a better understanding of both the promise of quantitative ultrasound as an assessment tool for muscle disorders and the known threats to measurement validity may foster greater adoption of this imaging modality in the management of muscular dystrophy and sarcopenia.

Since a distinction between normal age-related weakening of muscle strength and clinically significant muscle disease is not always obvious, the correct diagnosis is easily missed. Palmio and Udd (2014) performed magnetic resonance imaging in patients with three types of late onset limb girdle muscular dystrophy, highlighting differences as compared with a healthy aged-matched control. Therefore, muscle imaging should be considered as a means to distinguish between aging sarcopenia and muscle disease.

The quantitative and qualitative domains of sarcopenia concur to the development of the clinical phenotype, dominated by muscle weakness, poor balance, and slow gait speed. This clinical picture shows substantial overlap with that of physical frailty (Landi et al., 2015). The latter is a geriatric syndrome characterized by reduced homeostatic reserves, which exposes the individual at increased risk of negative health-related events in response to internal and/or external stressors. Based on the conceptual model proposed by Cesari et al. (2014), physical function represents the shared core of sarcopenia and physical frailty. Accordingly, the two conditions may be combined into one clinical entity. Such an operationalization provides researchers and clinicians with an objective, standardized, and clinically relevant condition that can easily be translated to the clinical arena. The recognition of skeletal muscle as the biological substrate of physical frailty is also functional to the design of new preventive and therapeutic interventions.

Merlini et al. (2014) evaluated the presence of sarcopenia and obesity in a cohort of 14 adult patients with muscular dystrophy. As determined by DXA, all of the patients resulted in sarcopenic based on appendicular lean mass index and obese according to the percentage of body fat. Skeletal muscle mass was markedly reduced in all patients and correlated with residual muscle strength, determined by hand-held dynamometry, and physical performance, as assessed by gait speed and respiratory function.

Toni et al. (2014) evaluated the nutritional status in seven patients with collagen type VI myopathies. As determined by DXA, all patients showed altered body composition. Specifically, all patients were sarcopenic, and all but one sarcopenic/obese. The authors found a negative correlation between basal energy expenditure per kilogram of fat free mass and the severity of the disease, which may be indicative of loss of muscular energetic efficiency.

Finally, treatments targeting sarcopenia with electrical stimulation (ES), nutritional intervention, and physical exercise were discussed.

The possibility of counteracting the age-related muscle decline was assessed through electrical stimulation (ES) of the thigh muscles (Kern et al., 2014). The effect of 9 weeks of training in healthy seniors was analyzed at functional, structural, and molecular levels. ES was able to improve muscle torque and functional performance, and increased the diameter of fast muscle fibers. At the molecular level, ES induced upregulation of IGF-1, the expression of markers of satellite cell activation, and downregulation of MuRF-1, a muscle specific atrophy-related gene. Conversely, autophagy was unaffected by ES.

The origins of sarcopenia are multifactorial and only partly understood. However, protein-energy malnutrition is a well-known cause of muscle loss in advanced age (Calvani et al., 2013). Noticeably, failure to meet an adequate dietary intake is also involved in the pathogenesis of osteoporosis. The coexistence of sarcopenia and osteoporosis greatly increases the risk of falls and fractures, respectively. The negative outcomes associated with geriatric fractures, especially of the hip, call for the development of novel strategies to reduce the incidence of new events and improve clinical and functional outcomes once the fracture has occurred. To this end, Calvani et al. (2014) explored the association between dietary intake and sarcopenia in a sample of older hip-fractured patients. As predicted, low dietary intake was found to be associated with reduced muscle mass at the time of hip fracture. This evidence provides the foundation for the design of nutritional interventions targeting the skeletal muscle to achieve therapeutic gain in this especially vulnerable patient population.

Besides the optimization of nutrition, engagement in regular physical activity is widely recognized as an effective measure for preventing and treating sarcopenia (Landi et al., 2014). As illustrated by Sanchis-Gomar et al. (2014), physical exercise is indeed able to modulate most of the pathways believed to underlie the pathogenesis of sarcopenia (e.g., cellular quality control mechanisms, sestrins, mitochondrial biogenesis, and oxidative stress). The authors also indicate a number of potential biological targets for drug development against sarcopenia. These include the age-dependent decrease in cellular NAD+ pool, the p16INK4a tumor suppressor, the FGF21-PGC-1α-irisin axis, the myostatin/follistatin pathway, and the IGF-1/Akt/mTOR axis.

## Conflict of Interest Statement

The authors declare that the research was conducted in the absence of any commercial or financial relationships that could be construed as a potential conflict of interest.

## References

[B1] Alway S. E. Myers M. J. Mohamed J. S. (2014). Regulation of satellite cell function in sarcopenia. Front. Aging Neurosci. 6:246. 10.3389/fnagi.2014.00246 25295003 PMC4170136

[B2] Bernardi P. Bonaldo P. (2013). Mitochondrial dysfunction and defective autophagy in the pathogenesis of collagen VI muscular dystrophies. Cold Spring Harb. Perspect. Biol. 5, a011387. 10.1101/cshperspect.a011387 23580791 PMC3632061

[B3] Bonaldo P. Braghetta P. Zanetti M. Piccolo S. Volpin D. Bressan G. M. (1998). Collagen VI deficiency induces early onset myopathy in the mouse: an animal model for Bethlem myopathy. Hum. Mol. Genet. 7, 2135–2140. 10.1093/hmg/7.13.2135 9817932

[B4] Bonaldo P. Sandri M. (2013). Cellular and molecular mechanisms of muscle atrophy. Dis. Model Mech. 6, 25–39. 10.1242/dmm.010389 23268536 PMC3529336

[B5] Calvani R. Martone A. M. Marzetti E. Onder G. Savera G. Lorenzi M. (2014). Pre-hospital dietary intake correlates with muscle mass at the time of fracture in older hip-fractured patients. Front. Aging Neurosci. 6:269. 10.3389/fnagi.2014.00269 25477815 PMC4236534

[B6] Calvani R. Miccheli A. Landi F. Bossola M. Cesari M. Leeuwenburgh C. (2013). Current nutritional recommendations and novel dietary strategies to manage sarcopenia. J. Frailty Aging 2, 38–53. 26082911 PMC4465574

[B7] Cesari M. Landi F. Vellas B. Bernabei R. Marzetti E. (2014). Sarcopenia and physical frailty: two sides of the same coin. Front. Aging Neurosci. 6:192 10.3389/fnagi.2014.00192 25120482 PMC4112807

[B8] Chakkalakal J. Brack A. (2012). Extrinsic regulation of satellite cell function and muscle regeneration capacity during aging. J. Stem Cell Res. Ther. S11, 001. 10.4172/2157-7633.S11-001 24678443 PMC3965255

[B9] De Palma C. Perrotta C. Pellegrino P. Clementi E. Cervia D. (2014). Skeletal muscle homeostasis in duchenne muscular dystrophy: modulating autophagy as a promising therapeutic strategy. Front. Aging Neurosci. 6:188. 10.3389/fnagi.2014.00188 25104934 PMC4109521

[B10] Decary S. Hamida C. B. Mouly V. Barbet J. P. Hentati F. Butler-Browne G. S. (2000). Shorter telomeres in dystrophic muscle consistent with extensive regeneration in young children. Neuromuscul. Disord. 10, 113–120. 10.1016/S0960-8966(99)00093-0 10714586

[B11] Deschenes M. R. (2011). Motor unit and neuromuscular junction remodeling with aging. Curr. Aging Sci. 4, 209–220. 10.2174/1874609811104030209 21529328

[B12] Eisenberg I. Alexander M. S. Kunkel L. M. (2009). miRNAS in normal and diseased skeletal muscle. J. Cell Mol. Med. 13, 2–11. 10.1111/j.1582-4934.2008.00524.x 19175696 PMC3072056

[B13] Fisher A. L. (2004). Of worms and women: sarcopenia and its role in disability and mortality. J. Am. Geriatr. Soc. 52, 1185–1190. 10.1111/j.1532-5415.2004.52320.x 15209660

[B14] Gattazzo F. Molon S. Morbidoni V. Braghetta P. Blaauw B. Urciuolo A. (2014). Cyclosporin A promotes in vivo myogenic response in collagen VI-deficient myopathic mice. Front. Aging Neurosci. 6:244. 10.3389/fnagi.2014.00244 25309428 PMC4163991

[B15] Goodpaster B. H. Park S. W. Harris T. B. Kritchevsky S. B. Nevitt M. Schwartz A. V. (2006). The loss of skeletal muscle strength, mass, and quality in older adults: the health, aging and body composition study. J. Gerontol. A Biol. Sci. Med. Sci. 61, 1059–1064. 10.1093/gerona/61.10.1059 17077199

[B16] Harris-Love M. O. Monfaredi R. Ismail C. Blackman M. R. Cleary K. (2014). Quantitative ultrasound: measurement considerations for the assessment of muscular dystrophy and sarcopenia. Front. Aging Neurosci. 6:172 10.3389/fnagi.2014.00172 25071570 PMC4094839

[B17] Hepple R. T. (2014). Mitochondrial involvement and impact in aging skeletal muscle. Front. Aging Neurosci. 6:211. 10.3389/fnagi.2014.00211 25309422 PMC4159998

[B18] Holland A. Dowling P. Ohlendieck K. (2014). New pathobiochemical insights into dystrophinopathy from the proteomics of senescent mdx mouse muscle. Front. Aging Neurosci. 6:109. 10.3389/fnagi.2014.00109 24917816 PMC4042888

[B19] Holmberg J. Alajbegovic A. Gawlik K. I. Elowsson L. Durbeej M. (2014). Laminin alpha2 chain-deficiency is associated with microrna deregulation in skeletal muscle and plasma. Front. Aging Neurosci. 6:155. 10.3389/fnagi.2014.00155 25071564 PMC4080261

[B20] Jungbluth H. Gautel M. (2014). Pathogenic mechanisms in centronuclear myopathies. Front. Aging Neurosci. 6:339. 10.3389/fnagi.2014.00339 25566070 PMC4271577

[B21] Kern H. Barberi L. Lofler S. Sbardella S. Burggraf S. Fruhmann H. (2014). Electrical stimulation counteracts muscle decline in seniors. Front. Aging Neurosci. 6:189. 10.3389/fnagi.2014.00189 25104935 PMC4109438

[B22] Krause S. (2015). Insights into muscle degeneration from heritable inclusion body myopathies. Front. Aging Neurosci. 7:13. 10.3389/fnagi.2015.00013 25729363 PMC4325924

[B23] La Rovere R. M. Quattrocelli M. Pietrangelo T. Di Filippo E. S. Maccatrozzo L. Cassano M. (2014). Myogenic potential of canine craniofacial satellite cells. Front. Aging Neurosci. 6:90. 10.3389/fnagi.2014.00090 24860499 PMC4026742

[B24] Landi F. Calvani R. Cesari M. Tosato M. Martone A. M. Bernabei R. (2015). Sarcopenia as the biological substrate of physical frailty. Clin. Geriatr. Med. 31, 367–374. 10.1016/j.cger.2015.04.005 26195096

[B25] Landi F. Marzetti E. Martone A. M. Bernabei R. Onder G. (2014). Exercise as a remedy for sarcopenia. Curr. Opin. Clin. Nutr. Metab. Care 17, 25–31. 10.1097/MCO.0000000000000018 24310054

[B26] Lim J. A. Li L. Raben N. (2014). Pompe disease: from pathophysiology to therapy and back again. Front. Aging Neurosci. 6:177. 10.3389/fnagi.2014.00177 25183957 PMC4135233

[B27] Malatesta M. Cardani R. Pellicciari C. Meola G. (2014). RNA transcription and maturation in skeletal muscle cells are similarly impaired in myotonic dystrophy and sarcopenia: the ultrastructural evidence. Front. Aging Neurosci. 6:196 10.3389/fnagi.2014.00196 25126079 PMC4115624

[B28] Marzetti E. Calvani R. Cesari M. Buford T. W. Lorenzi M. Behnke B. J. (2013). Mitochondrial dysfunction and sarcopenia of aging: from signaling pathways to clinical trials. Int. J. Biochem. Cell Biol. 45, 2288–2301. 10.1016/j.biocel.2013.06.024 23845738 PMC3759621

[B29] Marzetti E. Lorenzi M. Antocicco M. Bonassi S. Celi M. Mastropaolo S. (2014). Shorter telomeres in peripheral blood mononuclear cells from older persons with sarcopenia: results from an exploratory study. Front. Aging Neurosci. 6:233. 10.3389/fnagi.2014.00233 25221511 PMC4147848

[B30] Merlini L. Vagheggini A. Cocchi D. (2014). Sarcopenia and sarcopenic obesity in patients with muscular dystrophy. Front. Aging Neurosci. 6:274. 10.3389/fnagi.2014.00274 25339901 PMC4188124

[B31] Palmio J. Udd B. (2014). Borderlines between sarcopenia and mild late-onset muscle disease. Front. Aging Neurosci. 6:267. 10.3389/fnagi.2014.00267 25324776 PMC4179539

[B32] Pozzobon M. Franzin C. Piccoli M. De Coppi P. (2014). Fetal stem cells and skeletal muscle regeneration: a therapeutic approach. Front. Aging Neurosci. 6:222. 10.3389/fnagi.2014.00222 25221507 PMC4145352

[B33] Raz Y. Raz V. (2014). Oculopharyngeal muscular dystrophy as a paradigm for muscle aging. Front. Aging Neurosci. 6:317. 10.3389/fnagi.2014.00317 25426070 PMC4226162

[B34] Rosenberg I. H. (1989). Summary comments. Am. J. Clin. Nutr. 50, 1231–1233.

[B35] Rudolf R. Khan M. M. Labeit S. Deschenes M. R. (2014). Degeneration of neuromuscular junction in age and dystrophy. Front. Aging Neurosci. 6:99. 10.3389/fnagi.2014.00099 24904412 PMC4033055

[B36] Sabatelli P. Castagnaro S. Tagliavini F. Chrisam M. Sardone F. Demay L. (2014). Aggresome-autophagy involvement in a sarcopenic patient with rigid spine syndrome and a p.C150R Mutation in FHL1 Gene. Front. Aging Neurosci. 6:215. 10.3389/fnagi.2014.00215 25191266 PMC4137286

[B37] Sakuma K. Aoi W. Yamaguchi A. (2014). The intriguing regulators of muscle mass in sarcopenia and muscular dystrophy. Front. Aging Neurosci. 6:230. 10.3389/fnagi.2014.00230 25221510 PMC4148637

[B38] Sanchis-Gomar F. Pareja-Galeano H. Mayero S. Perez-Quilis C. Lucia A. (2014). New molecular targets and lifestyle interventions to delay aging sarcopenia. Front. Aging Neurosci. 6:156 10.3389/fnagi.2014.00156 25071565 PMC4078253

[B39] Tamaki T. Hirata M. Uchiyama Y. (2014). Qualitative alteration of peripheral motor system begins prior to appearance of typical sarcopenia syndrome in middle-aged rats. Front. Aging Neurosci. 6:296. 10.3389/fnagi.2014.00296 25400579 PMC4214197

[B40] Toni S. Morandi R. Busacchi M. Tardini L. Merlini L. Battistini N. C. (2014). Nutritional status evaluation in patients affected by Bethlem myopathy and ullrich congenital muscular dystrophy. Front. Aging Neurosci. 6:315. 10.3389/fnagi.2014.00315 25477818 PMC4235079

[B41] Zulian A. Tagliavini F. Rizzo E. Pellegrini C. Sardone F. Zini N. (2014). Melanocytes from patients affected by Ullrich congenital muscular dystrophy and Bethlem myopathy have dysfunctional mitochondria that can be rescued with cyclophilin inhibitors. Front. Aging Neurosci. 6:324. 10.3389/fnagi.2014.00324 25477819 PMC4238408

